# Semi-Quantitative Method for Streptococci Magnetic Detection in Raw Milk

**DOI:** 10.3390/bios6020019

**Published:** 2016-04-27

**Authors:** Carla Duarte, Tiago Costa, Carla Carneiro, Rita Soares, Andrei Jitariu, Susana Cardoso, Moisés Piedade, Ricardo Bexiga, Paulo Freitas

**Affiliations:** 1 INESC–MN Instituto de Engenharia de Sistemas e Computadores—Microsistemas e Nanotecnologias, Rua Alves Redol 9, 1000-029 Lisbon, Portugal; ritasoares18@hotmail.com (R.S.); ajitariu@phys-iasi.ro (A.J.); scardoso@inesc-mn.pt (S.C.); Paulo.Freitas@inl.int (P.F.); 2 CIISA at Faculdade de Medicina Veterinária, Universidade de Lisboa, Av. da Universidade Técnica, 1300-477 Lisbon, Portugal; carla.n.carneiro@gmail.com (C.C.); ricardobexiga@fmv.ulisboa.pt (R.B.); 3 INESC-ID Instituto de Engenharia de Sistemas e Computadores—Investigação e Desenvolvimento, Rua Alves Redol 9, 1000-029 Lisbon, Portugal; tiagomlcosta@gmail.com (T.C.); msp@inesc-id.pt (M.P.); 4 Visiting from the National Institute of Research and Development for Technical Physics, 47 Mangeron Blvd, Iasi 700050, Romania; 5 Instituto Superior Técnico, Physics Department, Av. Rovisco Pais, 1049-001 Lisbon, Portugal; 6 International Iberian Nanotechnology Laboratory (INL), Av. Mestre José Veiga, 4715-330 Braga, Portugal

**Keywords:** magnetoresistive sensors, magnetic nanoparticle (NP), *Streptococcus agalactiae*, *Streptococcus uberis*, milk, immunogenic recognition, microfluidic

## Abstract

Bovine mastitis is the most costly disease for dairy farmers and the most frequent reason for the use of antibiotics in dairy cattle; thus, control measures to detect and prevent mastitis are crucial for dairy farm sustainability. The aim of this study was to develop and validate a sensitive method to magnetically detect *Streptococcus agalactiae* (a Group B streptococci) and *Streptococcus uberis* in raw milk samples. Mastitic milk samples were collected aseptically from 44 cows with subclinical mastitis, from 11 Portuguese dairy farms. Forty-six quarter milk samples were selected based on bacterial identification by conventional microbiology. All samples were submitted to PCR analysis. In parallel, these milk samples were mixed with a solution combining specific antibodies and magnetic nanoparticles, to be analyzed using a lab-on-a-chip magnetoresistive cytometer, with microfluidic sample handling. This paper describes a point of care methodology used for detection of bacteria, including analysis of false positive/negative results. This immunological recognition was able to detect bacterial presence in samples spiked above 100 cfu/mL, independently of antibody and targeted bacteria used in this work. Using PCR as a reference, this method correctly identified 73% of positive samples for streptococci species with an anti-*S. agalactiae* antibody, and 41% of positive samples for an anti-GB streptococci antibody.

## 1. Introduction

Bovine mastitis, the inflammation of the mammary gland most often with infectious origin, is the most costly disease for dairy farmers and the most frequent reason for the use of antibiotics in dairy cattle; thus, control measures to prevent mastitis are crucial for farm sustainability. The identification of contagious bacteria that cause mastitis is necessary to control the disease in the herd, reduce the risk of chronic infections, and target antimicrobial therapy. *Streptococcus agalactiae* (a Lancefield Group B Streptococci) and *Streptococcus uberis* (no Lancefield group) are major mastitis pathogens [1] that can be transmitted from cow to cow in the milking parlor in a contagious way [2]. Their identification is currently performed most often through conventional bacteriology, by growth of bacteria in culture media, isolation, and identification based on phenotypic features. This methodology is time-consuming, with results taking between 48 and 72 h to be obtained, and can lead to no-growth results corresponding to false negatives. In these cases, phenotypic identification is being supplemented with genotypic methods, such as polymerase chain reaction (PCR) [3], for more accurate identification of bacteria associated with intramammary infections.

The suitability of a detection method for routine diagnosis as “cow-side use” depends mainly on the time to produce results, sensitivity, and specificity. Immunological identification of mastitis pathogens has been reported [4,5]. These authors suggested that the diagnosis of clinical mastitis cases could be considerably enhanced if samples showing no growth on culture media could be subjected to an enzyme-linked immunosorbent assay (ELISA), because of the antibodies' observed ability to detect soluble, as well as insoluble, antigens, independently of intact bacterial cell presence in milk. The basis for a true positive result in immunological analysis is the confidence on the specificity of the selected antibody. As mentioned in previous work [6], Western blotting assays using a polyclonal anti-GB streptococci antibody evidenced two stained immunogenic proteins in *Streptococcus uberis* cell wall proteins’ pattern besides the expected immunogenic protein set of *Streptococcus agalactiae*. According to Groschup and colleagues [7], some *Streptococcus uberis* strains can also react with Lancefield group B serum.

The use of portable platforms to detect bacteria has been optimized [8] allowing for cell separation, identification and counting to be achieved in a compact and modular format. This feature can be combined with magnetic detection, where magnetoresistive (MR) sensors can be integrated within microfluidic channels to detect magnetically-labeled cells, being promising as one emerging technology for magnetic biodetection [9,10].

The aim of this study was to develop and validate a sensitive method for magnetic detection of *Streptococcus agalactiae* and *Streptococcus uberis* in raw milk samples. For both magnetic detection and conventional microbiology methods, sensitivity, specificity, and positive predictive value (PPV) were calculated in comparison with the PCR reference method.

## 2. Materials and Methods 

### 2.1. Method Principles

**Figure 1:**
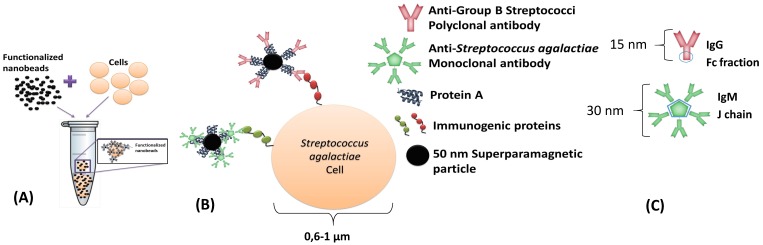
Schematic of immuno-magnetic detection of cells. (**A**) Incubation of functionalized NPs with bacterial cells; and (**B**) biological affinities between different functionalized NPs with bacterial cell wall immunogenic proteins; (**C**) Predictable protein A binding site to each antibody.

This dynamic detection is based on the detection of the fringe field created by magnetic particles attached to the bacterial cells. By selecting the suitable antibodies, it is possible to perform immunological recognition of Group B Streptococci (including *Streptococcus agalactiae*) and of *Streptococcus uberis* immunogenic proteins (Figure 1A,B). A polyclonal anti-GB Streptococci IgG (8435-2000, AbD Serotec, Kidlington, UK) and one monoclonal anti-*Streptococcus agalactiae* IgM (MA1-10871, Thermo Fisher, Waltham, USA), were used separately. The antibodies were expected to attach to protein A of Nanomag^®^-d-spio 50 nm particles (79-20-501, MicromodPartikeltechnolo-gie GmbH, Rostock, Germany), by the Fc fraction in immunoglobulins G and by the joining chain (J chain) in immunoglobulins M (Figure 1C). Antibodies and bacterial cell dimensions are shown in Figure 1.

The bio-functionalization of nanoparticles was achieved by the addition of 7.27 μL from the nanoparticles’ original vial to 0.53 μL of polyclonal anti-GB streptococci antibody (1 mg/mL) (or to 5.5 μL of monoclonal anti-*Streptococcus agalactiae* antibody (0.5 mg/mL)) in 492.2 µL (or 487.2 µL) of PBS. The incubation step required 1 h at room temperature (RT) and continuous agitation. Final functionalized particles were magnetically isolated by a MS column (130-042-201 Miltenyi, Bergisch Gladbach, Germany) according to MACS MiltenyiBiotec protocol and eluted with phosphate-buffered saline (PBS) + 0.5% bovine serum albumin (BSA) + 2mM ethylene diamine tetra acetic acid (EDTA) buffer after removal of the MS column from the magnet. A volume of 2 µL of this final suspension was added to each PBS or milk sample.

### 2.2. Biosensor Fabrication

**Figure 2:**
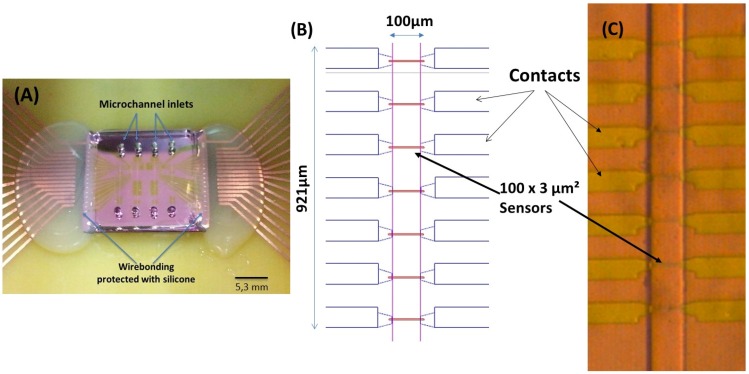
(**A**) Final device with the magnetoresistive chip bonded to the polydimethylsiloxane (PDMS) microchannels. The sensor’s wirebonding are protected with silicone. (**B**) Spinvalve (SV) sensor distribution along the microchannels; and (**C**) microscope photo of the fabricated SVs with the PDMS microchannel over them (20× amplification).

Following a previously reported work for magnetic particle detection [10,11], an integrated cytometer platform was used, consisting of magnetoresistive sensors, readout/acquisition electronics and a microfluidic channel where the milk was injected. The device geometry and physical principles of operation are described in Fernandes [6], and are based on spin valves (SV) deposited by ion beam deposition on a Nordiko 3000 tool with the following structure: Si/Al_2_O_3_ 60/Ta1.5/Ni_80_Fe_20_ 2.5/Co_90_Fe_10_ 2.0/Cu 2.1/Co_90_Fe_10_ 2.0/Mn_76_Ir_24_ 6.0/Ta 5.0 [12,13] (thickness in nm, compositions in atomic %), patterned with 3 μm × 100 μm active dimensions (measured between the AlSiCu 300 nm thick contact leads), according to Figure 2. Passivation was done with a 300 nm thick Si_3_N_4_ layer deposited by PECVD (Electrotech Delta chemical vapor deposition system). Sensors were annealed at 250 °C for 15 min, in vacuum, and cooled under a 1 Tesla magnetic field.

The SV sensors’ electrical transport characterization (resistance *versus* DC magnetic field) provided information on the magnetoresistance, defined as MR = (R_max_ − R_min_)/R_min_ (where R_max_ and R_min_ are the maximum and minimum resistance levels). The sensor sensitivity is defined as the slope of the curve over the linear range of operation and ranges 0.15%–0.17%/Oe for the sensors measured across the wafer.

The magnetic detection mechanism used nanoparticles which had a superparamagnetic signature, therefore requiring an external magnetic field to activate their magnetization. This was done with an external vertical field created by a permanent magnet (NdFeB, 20-10-01STIC, Supermagnete, Gottmadingen, Germany) mounted below the printed circuit board (PCB). After magnet alignment below this sensor, the effect of the small components in the plane of the sensors was visible in their sensitivity decrease to 0.074%/Oe. The magnetic field strength at the microfluidic channel center was ~31 mT, so the individual nanoparticles were magnetized with a magnetic moment of 2.0 × 10^−18^ A·m^2^. Upon magnetization, the nanoparticles created a magnetic field fringe field at the sensor surface; therefore, the particle presence was detected through the changes in the sensor resistance (or voltage, as shown in Figure 5).

### 2.3. Microfluidic Channel Fabrication

The microchannels (Figure 2B) were fabricated in polydimethylsiloxane (PDMS), with 100 μm (length) × 50 μm (height), following the method described in Fernandes and colleagues’ work [6].

The integration of the magnetoresistive chip with the PDMS microchannels was achieved through irreversible bonding of the Si_3_N_4_ and PDMS surfaces. Both surfaces were exposed to ultraviolet/ozone (UVO Cleaner, Jelight Company Inc., Irvine, CA, USA) for 15 min and then mounted face-to-face and manually aligned to be kept at RT, overnight. The ensemble was then mounted in a PCB, where the sensors were wire-bonded and the wires protected with silicone (Figure 2A).

The raw milk samples’ constant flowing through the microchannel section of 50 µm height and 100 µm length was challenging because of its density and colloidal behavior. A surfactant addition to milk samples, namely Tween 20, was used to achieve higher dispersion of fat globules (0.1–10 µm), allowing lower interfacial tension and its dimension reduction [14]. On the other hand, we adopted the milk preparation method of dairy industries for milk homogenization, using agitation (vortex) and higher temperatures (60 °C) to decrease fat globules’ dimensions and allow their uniform distribution in the sample.

### 2.4. Readout Electronics

The multi-channel PCB designed to interface 15 spin valve sensors was connected to an amplifier with an operating gain of 5000×, and high-pass and low-pass filters of 300 Hz and 10 kHz, respectively. Each channel included a configurable DC current source, from 0.25 mA to 2 mA [15].

**Figure 3:**
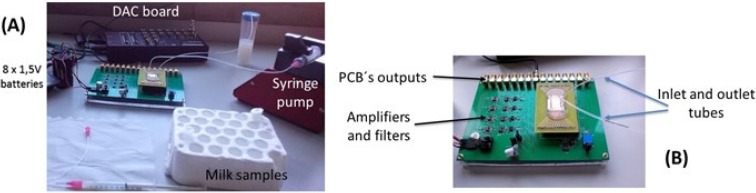
(**A**) Acquisition setup assembly; and (**B**) multi-channel PCB connected to external electronics.

In this work, only one sensor per channel was monitored. One syringe pump was attached to the system, and was the only device not operating under DC batteries (thus, introducing the 50 Hz noise from the main power grid). The sensor output signals were recorded over time by using a connection to an acquisition setup composed by a 16 bit analog-to-digital converter (ADC board DT9836-12-2-BNC), at 50 kHz acquisition frequency. The resulting digital signals were then post-processed in a software developed in Matlab, to apply a low pass digital filter with cut-off frequency of 2 kHz, allowing real-time noise characterization and data-storing into the hard drive for further analysis (Figure 3). The sensors used for this work showed a noise level of 2.5–4 µV (peak-to-peak). During the experiments, the pump operation increased the noise level to 3–4.5 µV.

### 2.5. Magnetic Detection Method Calibration

A blank sample (only PBS or sterile raw milk) and a negative control sample (PBS or sterile raw milk, with 2 µL of functionalized NPs) were always measured prior to the measurements with contaminated milk, giving the background signal of the system.

A first calibration assay was then made with *Streptococcus agalactiae*/pAb anti-GB streptococci spiked on PBS sample.

Finally, *Streptococcus agalactiae*/pAb anti-GB streptococci; *Streptococcus uberis*/pAb anti-GB streptococci and *Streptococcus agalactiae*/mAb anti-*Streptococcus agalactiae* were spiked in sterile milk samples and the correspondent calibration curves made. Each concentration point was the result of three different assays’ measurements.

The calibration range between 0.1 and 20 cfu/µL was established taking into account the detection limit for conventional microbiology of 500 cfu/mL (0.5 cfu/µL).

### 2.6. Bacterial Cells

*Streptococcus agalactiae* (strain CECT 183) and *Streptococcus uberis* (strain CECT 994) cells were grown separately onto Columbia agar supplemented with 5% sheep blood (bioMérieux, 43021, Marcy l’Étoile, France) and incubated at 37 °C, overnight. A single colony of each isolate was selected and re-suspended in 4 mL of tripticase soy broth over 24 h at 37 °C. Subsequently, the bacterial cells were collected through centrifugation (15 min, 17 °C, 2700 rpm) and re-suspended in PBS (pH 7.2) to allow optical density measurement (at 600 nm) (BECKMAN DU-68 Spectrophotometer) and for colony-forming unit (cfu) estimation. A bacterial suspension with a known concentration of 10^4^ cfu/µL was the starting point to get seven different bacterial concentrations for each species, in PBS or in raw milk samples: 0.1; 0.3; 0.5; 1; 2; 10, and 20 cfu/µL.

### 2.7. Sterile Milk Samples

Raw milk used for the definition of calibration curves experiments was collected aseptically from healthy cows. Conventional microbiological tests were performed according to NMC protocols [16], to confirm no bacterial growth. Briefly, a raw milk sample (10 μL) was plated on a Columbia agar plate and on MacConkey agar plate (CM0007, Oxoid, Hampshire, UK) and both were incubated at 37 °C for 48 h. The absence of growth on both plates was considered to be equivalent to the presence of no viable bacteria in the milk.

Each sample for biosensor testing had a 500 µL volume consisting of 2 µL of a suspension of functionalized NPs, 98 µL of PBST, and 400 µL of one of seven bacterial suspensions with pre-defined bacterial concentrations in PBS or sterile raw milk. The incubation of these samples was performed at RT for 3 h, under agitation.

All raw milk samples were submitted to a pre-treatment of 15 min at 60 °C in a dry bath incubator (Grant, model QBD2, Leicestershire, UK) and 15 min of continuous centrifugation in a vortex mixer (Labnet International Inc., Edison, NJ, USA) after adding bacteria and PBST. Only then, 2 µL of functionalized NPs suspension were added for final incubation step.

### 2.8. Mastitic Milk Samples

Mastitic milk samples were collected aseptically from 44 cows originating in 11 Portuguese dairy farms. Animals were selected based on the presence of subclinical mastitis, defined by evidence of a somatic cell count over 1,000,000 cells/mL. Mammary quarters to sample were selected in these cows based on a strong positive reaction on the California Mastitis Test. Bacteria identification in mastitic milk samples was performed according to NMC protocols [16].

These mastitic milk samples were distributed in two groups of *n* = 31 samples each for biosensor analysis, of which 16 were tested by both antibodies (anti-GB streptococci and anti-*Streptococcus agalactiae*). The selection of quarter milk samples was based on bacteriological results which included *Staphylococcus aureus* (*n* = 1), *Streptococcus agalactiae* (*n* = 13), *Streptococcus uberis* (*n* = 11), *Streptococcus* spp. (*n* = 4), coagulase-negative staphylococci (CNS) (*n* = 2), *Enterococcus* spp. (*n* = 7), *Escherichia coli* (*n* = 3), Yeasts (*n* = 3) and *Prototheca* (*n* = 2). All mastitic milk samples were also submitted to PCR analysis.

### 2.9. PCR Reference Method Analysis

PCR analysis was performed by an external laboratory (VACUNEK, SL) with the PathoProof Mastitis Complete-16^®^ (Thermo Scientific), a development of the PCR assay initially described in [17], which allows for the detection of 16 bovine mastitis pathogens. This method is semi-quantitative, classifying the amount of bacterial cells in mastitic milk samples as “high”, “average”, or “low”.

### 2.10. Biosensor Analysis

A volume of 400 µL of mastitic milk was collected and mixed with 98 µL of PBST. Each 498 µL sample was submitted to a pre-treatment of heating (15 min at 60 °C) and homogenization (15 min in vortex). After these steps, a volume of 2 µL of a functionalized NPs suspension was added to reach a final volume of 500 µL to be submitted to incubation (RT, 3 h, under agitation) and further biosensor analysis.

**Figure 4:**
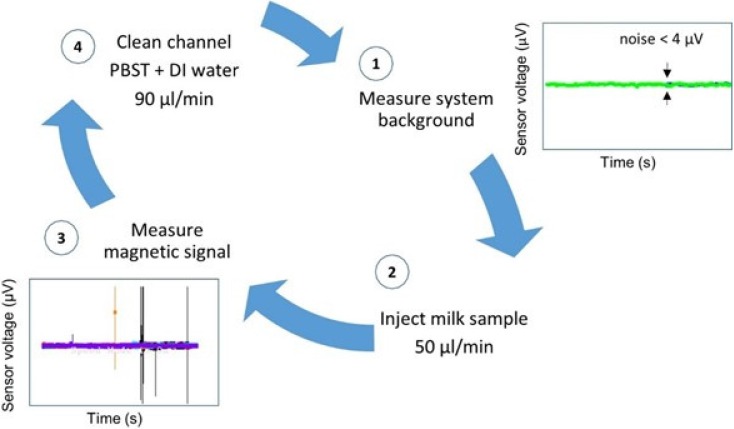
Biosensor analysis procedure steps.

Trials were performed with each antibody set consisting of 31 mastitic samples in different assays. Therefore, the biosensor analysis was validated 62 times. Each trial day began with sensor’s noise level measurement (Figure 4-1) and each sample was injected at a flow rate of 50 µL/min, through a PDMS microchannel (Figure 4-2). The microchannel was always cleaned between samples with PBST followed by deionized water, both at a 90 µL/min flow rate (Figure 4-4), until reaching noise level values again, denoting a magnetic-free microchannel filling. A blank sample and a negative control sample were measured whenever a new MR sensor was used and always before mastitic samples analysis.

**Figure 5:**
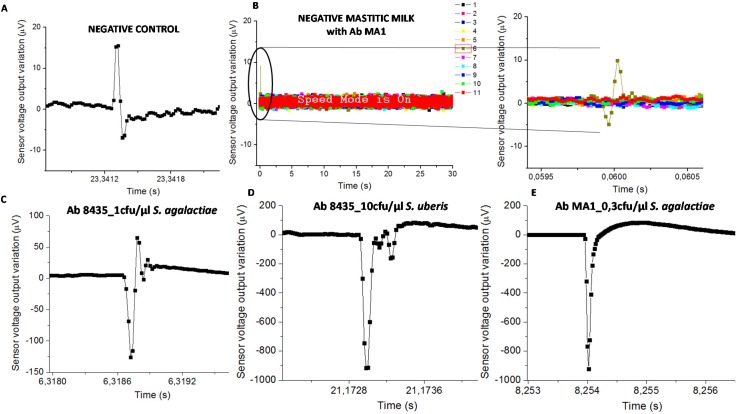
Sensor output for (**A**) negative control with the higher amplitude of 23 µV; (**B**) mastitic milk (without *S. agalactiae* according to the PCR) with NPs functionalized with mAb anti-*S. agalactiae* which presents an amplitude peak of 15 µV (9076AD sample code). The higher amplitude peaks found for each pair of bacteria-antibody were; (**C**) 193.6 µV in raw milk with anti-GB streptococci and 1 cfu/µL of *S. agalactiae*; (**D**) 917.5 µV in raw milk with anti-GB streptococci and 10 cfu/µL of *S. uberis*; and (**E**) 923.7 µV in raw milk with anti-*S. agalactiae* and 0.3 cfu/µL of *S. agalactiae*.

Samples with functionalized NPs on PBS or sterile milk (negative controls) evidenced signal less than 50 µV (Figure 5A). Mastitic milk samples without the targeted bacteria (proved by PCR) and tested with NPs functionalized with chosen antibodies, also evidenced signal less than 50 µV (Figure 5B). Samples used for calibration assays spiked with bacterial cells on sterile milk showed magnetic signal upper than 50 µV (Figure 5C–E).

The classification of mastitic milk samples by the biosensor was based on bacterial detection (presence or absence). Consequently, the “positive” samples were those with at least one magnetic peak above 50 µV, therefore higher than the signal found in negative control samples and in mastitic milk samples without targeted bacteria. Next, this “positive” sample magnetic peak should evidence a bipolar or unipolar shape similar to the ones found in samples used for calibration trials as shown in Figure 5C–E.

Biosensor analysis was a dynamic detection where a heterogeneous milk sample flowed inside the microchannel. Magnetically labelled bacterial cells were mixed randomly in milk leading to the impossibility of predicting its position above the sensor over time. Consequently, the magnetic peaks’ shape and time resulting from biosensor analysis were expected to be different between samples (Figure 5).

### 2.11. Data Analysis

Isolates were considered to be correctly identified by magnetic detection if the same species was found by the reference method, or if the magnetic detection did not identify the species it was targeting and PCR identified one of the other bacteria. For example, a correct identification referred to a *S. agalactiae* being identified magnetically in a sample that PCR had identified as *S. agalactiae*, but also when not identifying as *S. agalactiae* a sample that through PCR was identified as *Staphylococcus* spp. Regarding conventional microbiology, isolates were considered to be correctly identified if the same species was found as with the reference method. Misidentification was considered when the magnetic detection and conventional microbiology identified a different species than the reference method. For example, a sample that was identified as *Staphylococcus* spp. by PCR and that was identified as *S. agalactiae* by magnetic detection, or a sample that was identified as *S. agalactiae* by PCR and not identified as such by magnetic detection. For both magnetic detection and conventional microbiology methods, sensitivity, specificity, and positive predictive value were calculated in comparison with PCR species identification. Sensitivity was calculated as the proportion of the true positive isolates that were correctly identified with the magnetic detection or microbiological tests, e.g., the proportion of *S. agalactiae* isolates based on PCR analysis that were identified as such by magnetic detection and microbiology testing. Specificity was calculated as the proportion of the true negatives that were correctly identified with the magnetic detection and the microbiological tests, e.g., the proportion of isolates other than *S. agalactiae* based on PCR analysis that were identified as something other than *S. agalactiae* by magnetic detection and by microbiological testing. Finally, PPV was calculated as the proportion of isolates identified as a specific species based on magnetic detection or on microbiological testing that truly represented that particular species, e.g., the proportion of isolates that were identified as *S. agalactiae* by magnetic detection or microbiological testing that had been identified as *S. agalactiae* based on PCR analysis.

## 3. Results

### 3.1. Evaluation of Biosensor’s Bacterial Quantification

**Figure 6:**
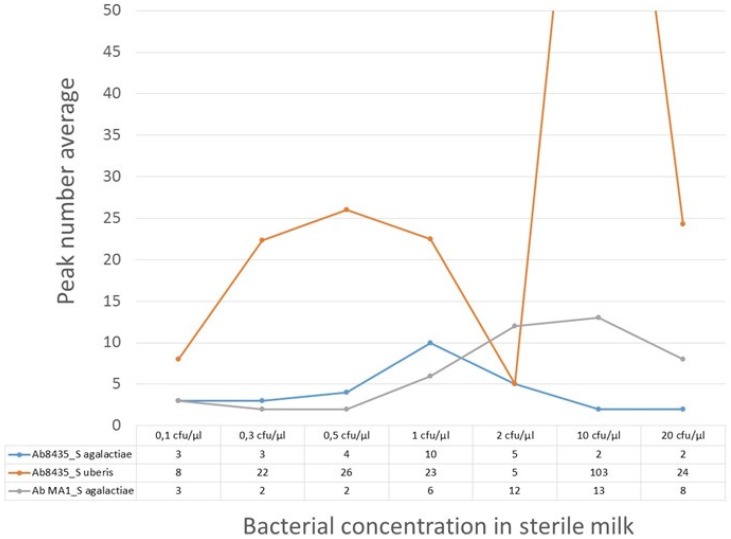
Calibration trial results for milk samples with seven bacterial concentrations (*S. agalactiae* or *S. uberis*) and functionalized NPs with pAb anti-GB streptococci (Ab8435) and mAb anti-*S.* *agalactiae* (Ab MA1). Peak number average for each bacteria-antibody pair are counted.

The calibration trial outcome is shown in Figure 6. The peak´s number per signal amplitude were calculated, showing no evidence of linear correlation with increasing bacterial concentration. The milk samples with the anti-GB streptococci antibody revealed the most exuberant signal with *S. uberis* when compared with the other two bacteria-antibody pairs (Figure 6). Only the *Streptococcus agalactiae*/pAb anti-GB streptococci pair showed no evidence of peaks higher than 200 µV. Despite that, these MR sensors could detect *Streptococcus agalactiae* and *Streptococcus uberis* in milk samples from 0.1 cfu/µL (100 cfu/mL).

**Figure 7:**
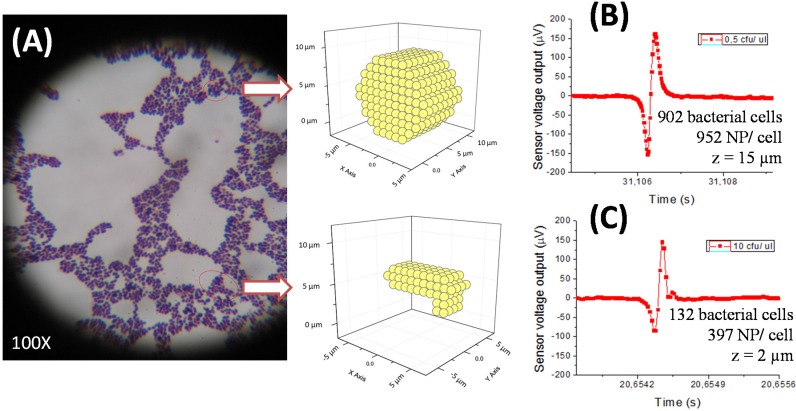
*Streptococcus agalactiae* cells microscopic image where a spherical cluster and an elongated cluster are evidenced (**A**). Experimental data fitting of the highest amplitude peaks obtained in PBS samples with different *S. agalactiae* concentrations (0.5 cfu/µL: 162 µV (**B**) and 10 cfu/µL: 146 µV (**C**)) during calibration curve settlement.

The calibration curve for PBS samples with bacteria was obtained for the *Streptococcus agalactiae*/pAb anti-GB streptococci pair. It showed evidence that different bacterial concentrations, as in sterile milk, also presented similar amplitude peaks (under 200 µV). Performing experimental data fitting to simulations for cell quantity estimation by peak, we obtained different results depending on considered functionalized NPs number per cell and cell cluster positioning above the MR sensor (height z) (Figure 7B,C).

### 3.2. Validation of Magnetic Detection

Forty-six mastitic milk samples with known bacteriology results, obtained through conventional microbiology were analyzed by PCR. A total of 160 identifications were performed by PCR for all 46 milk samples analyzed with mAb anti-*S. agalactiae* and mAb anti-GB streptococci. The most frequently isolated species based on the PCR were *Staphylococcus* spp., *E. coli*, and yeasts, followed by *S. uberis* and *S. agalactiae*. As a result of the high sensitivity of the PCR methodology, an average of four different pathogens were detected per mastitic milk sample, not allowing for the true causative agent of mastitis to be determined. Therefore, it was decided to use the conventional bacteriology results as the basis for the true identification, confirmed by the PCR (Table 1).

The magnetic detection with the anti-*Streptococcus agalactiae* antibody tested 31 mastitic milk samples from the total of 46 analyzed by conventional microbiology, which 10 and 13 were identified as *S. agalactiae* (Table 1), respectively. However, from these 31 samples tested, 11 were identified as *S. agalactiae* by PCR and only one was not identified as such by microbiology, but as *S. uberis*. The magnetic detection with the anti-*S. agalactiae* antibody identified 8 *S. agalactiae* isolates correctly in 11 (72.7%) milk samples with this species when compared to PCR. Five mastitic milk samples did not lead to an identification by this polyclonal antibody because they did not present *S. agalactiae* according to the PCR analysis (true negatives) (Table 2). Misidentification was observed for 18 of 31 (58%) isolates in the 31 mastitic milk samples tested with the anti-*Streptococcus agalactiae* antibody (Table 1). Only three misidentified *S. agalactiae* isolates were found in milk samples with this species analyzed by PCR, evidencing a failure of recognition by this monoclonal antibody (Table 1). Adding to that, five mastitic milk samples with *S. uberis* and/or *S. dysgalactiae* and 10 mastitic milk samples without any Streptococci species according to the PCR analysis, were misidentified by biosensor analysis as having *S. agalactiae* and were all classified as false positives (Table 2). Overall a 73% sensitivity, 25% specificity, and 35% PPV were found for magnetic detection with the anti-*Streptococcus agalactiae* antibody. The highest sensitivity value represents the proportion of the true positives (8) that were correctly identified with this monoclonal antibody (Table 2).

Using the polyclonal anti-GB streptococci in magnetic detection, the 31 mastitic samples tested included 16 identified equitably as eight *S. agalactiae* and eight *S. uberis* by conventional microbiology (Table 1). However, PCR analysis identified two more samples as *S. uberis* in the 31 analyzed by this antibody, amounting 18 bacterial target possibilities. The magnetic detection with the anti-GB streptococci antibody correctly identified seven streptococci isolates present in 18 (38.9%) milk samples with *S. agalactiae* and/or *S. uberis* according to PCR analysis. The microorganisms that were not identified as GB streptococci or *S. uberis* (13/31) by the reference method in mastitic milk samples, were magnetically detected as GB streptococci and/or *S. uberis* in those samples (five false positives) or else, undetected as true negatives (8) (Table 2). Misidentification was observed for 16 isolates in the 31 (51.6%) mastitic samples tested. Eleven misidentified *S. agalactiae* and *S. uberis* isolates were found in milk samples analyzed by PCR with these two streptococci, evidencing a failure of recognition by this polyclonal antibody (Table 1). Overall a sensitivity of 41%, a specificity of 57%, and a PPV of 54% were found for magnetic detection with the anti-GB streptococci antibody. The highest specificity value represents the proportion of the true negatives (8) that were correctly identified with this polyclonal antibody (Table 2).

With regards to microbiological testing for all 46 samples considered, the highest agreement in microorganism identification, in comparison with PCR analysis, was found to be 100% for *S. agalactiae*, *Staphylococcus* spp., *E. coli*, and *Prototheca*, as showed in percentage data of correct identification (Table 1). However, incomplete microbiological identifications of 67.4% (31/46) and a misidentification of 32.6% (15/46) were observed (Table 1). Microbiological tests evidenced a PPV value of 67% and a sensitivity of 100% to identify mastitis pathogens in milk samples, showing that conventional microbiology correctly identified true negatives (Table 2).

## 4. Discussion

Sensitivities of 73% and 41% and specificity values of 25% and 57% were obtained for magnetic identification of streptococci species with an anti-*Streptococcus agalactiae* antibody and an anti-GB streptococci antibody, respectively. The higher PPV value (54%) evidenced for magnetic detection with the anti-GB streptococci antibody may reinforce the bonding avidity between this polyclonal antibody and the only two immunogenic cell wall proteins of *S. uberis*, compared to 10 or more antigens known in *S. agalactiae*.

Comparing sensitivity and specificity values of this magnetic detection with another study that used immunological detection of mastitis pathogens through an ELISA for detecting *S. aureus* in milk [18], higher sensitivity (69%–90%) and specificity values (61%–97%) [4] were observed. ProStaph test (Proscience Corp, Albertson, NY, USA) had a detection limit of 10^4^–10^5^ cfu/mL, when the minimum bacterial presence detected by the present immunological recognition was 100 cfu/mL, independently of the antibody and targeted bacteria used.

The microbiological misidentification ratio of 32.6% observed in our study was due to 15 wrong identifications compared with PCR analysis. The use of PCR for the identification of mastitis pathogens may have the advantage [19] of leading to decreased false negative results, but it may also be clinically challenging. PCR’s higher sensitivity leads to the identification of all milk sample pathogens and contaminants alike [20], being difficult to assign mastitis causality to a particular microorganism. This was also observed in our study, with the average number of microorganisms identified per milk sample being one.

Regarding the validation of the magnetic detection method, some false positive results could be explained by NPs’ agglomeration by the mastitic milk matrix heterogeneity, sporadic low cleaning efficiency of the channel’s inlet chamber, and also due to electrical conductivity of mastitic milk samples. Bovine mastitis leads to changes in ion concentration of milk due to increased vascular permeability, resulting from inflammatory response, leading to modifications in electrical conductivity of milk [21]. The conductance in milk causes sensor’s resistivity variation translated by higher background noise. Despite a detergent (Tween 20) being in milk samples to reduce fat globules dimensions, to distribute the bacterial cells in the milk, to improve nanoparticles mobilization and to allow for a more homogeneous milk matrix, the optimization trials (not described in this paper) showed the need for a compromise between Tween 20 quantity and magnetic peak discrimination. Different volumes of PBST in 500 µL of milk samples were used. The higher volumes (>100 µL) showed evidence of bubbles inside the microchannel which hampered milk flowing, caused nanoparticle agglomeration, and did not help magnetic peak discrimination between control samples (milk with only NPs) and samples with bacteria. On the other hand, false negatives may have occurred because of three circumstances. Firstly, the binding yield variations between antibodies and NPs and/or failure in bacterial cells magnetic labelling could narrow bacterial cells identification. This fact should be recognized as possible because IgM and nanoparticles dimensions are closer, so it will be more difficult to have the same number of attached IgM when comparing with NPs functionalization with smaller IgG. Secondly, according to Henriksen’s study [22], it is possible that the rotating nature of the magnetic dipole field of NPs magnetized by an external magnetic field, can induce signal cancelation. Therefore, the fields from two differently-placed NPs can partially cancel each other. Finally, microchannel current height (50 µm) could be reduced to improve sensor’s detection by forcing bacterial cells dragging over it, but mastitic milk trials showed that milk clots hamper sample flow and height decrease leads to microchannel obstruction, pointing to a compromise between the sensor’s detection and sample fluidity.

With regard to bacterial quantification data, this magnetic detection method showed some microbiological and immunological constraints. Bacterial cells group together randomly depending on growth conditions [23]. Each bacteria may express a different number of cell wall proteins, including the immunogenic ones [24]. Together, these facts limit the knowledge of how many immunogenic proteins there are per cell and consequently, how many proteins will be recognized by each specific antibody used. On the other hand, the chemical and colloidal changes of milk components in a state of inflammatory response [14], as occurs with mastitis, prevent and reduce bacterial magnetic labeling efficacy [25]. Consequently, it was not possible to predict peak profiles (number, shape) for each bacterial concentration.

Although the sensitivity of the magnetic detection method is important, many additional factors must be considered, including rapidity, easy to use, flexibility, portability, and costs [26]. This dynamic methodology showed it was possible for a mastitic milk sample to be processed until a final result was obtained in five hours, but was not suitable for processing a large number of samples (maximum of 10–12 per day). It also showed technical simplicity when established, and ease of scoring and interpreting the results. Despite the lower sensitivities obtained, both antibodies used were capable to detect bacterial cells in real milk samples. However, other antibodies could be used for further identification of different bovine mastitis pathogens, reinforcing this method flexibility.

Further studies could be done for biosensor’s performance improvement as higher number of analyzed samples per day by using all seven SV of each microchannel and use all of them per die. Another opportunity for better bacterial magnetic signal acquisition could be the dilution of milk samples in water or bacterial isolation from mastitic milk to be further analyzed in PBS, reducing conductance problems.

## 5. Conclusions

A lab-on-a-chip magnetoresistive cytometer, with microfluidic sample handling, was successfully used to demonstrate the minimal detection of 100 cfu/mL of bacterial cells in raw milk. Milk samples were mixed with a solution combining specific antibodies and magnetic nanoparticles, before the analysis. This paper describes the methodology used for detection of bacteria, including analysis of false positive/negative results.

Comparison with PCR results showed sensitivities of 73% and 41%, specificity values of 25% and 57%, and PPV values of 35% and 54% for magnetic identification of streptococci species with an anti-*S. agalactiae* antibody and an anti-GB streptococci antibody, respectively.

Magnetic detection of milk samples showed some microbiological and immunological constraints. Since bacterial cells have high variability on the number of immunogenic proteins per cell, the number of labeled sites through the antibody is also not well defined. This affects the quantification of the magnetic method. As a consequence, it was not possible to quantify the peak profile (number, shape) for each bacterial concentration. The method, however, allows the determination of their presence, and quantification may be done within lower/upper threshold limits. Simulations of the sensor output as a function of the nanoparticle distribution over the cells (using colonies/clusters configurations compatible with the experimentally observed under microscopy) can provide an indication of minimum and maximum numbers. Further work should be done towards a more accurate quantification based on simulations.

Accuracy in bacterial quantification was affected by false positive results, leading to overestimation of bacteria numbers caused by nanoparticle agglomeration by the mastitic milk matrix heterogeneity and/or microchannel blocking. Additionally, undetected bacteria due to false negatives may be due to binding yield variations between antibodies and nanoparticles and/or failure in bacterial cells magnetic labelling.

This biosensor can be submitted to further improvements which may include incorporation of a milk pre-treatment step into the microfluidic platform and also further studies on electronics to allow multiplex analysis of several samples at the same time. Currently, this biosensor requires an external computer for system operation and displaying test results, so a fully integrated system into a single device could also be made.

## Figures and Tables

**Table 1:** Identification of isolates in mastitic milk samples with both magnetic detection (mAb anti-*Streptococcus agalactiae*; pAb anti-GB streptococci) and with conventional microbiology, compared to PCR analysis as the reference method.

		Magnetic Detection						Microbiological Tests		
		Anti-*Streptococcus agalactiae*			Anti-GB Streptococci					
Mastitic Milk Isolates		Correctly Identified			Correctly Identified			Correctly Identified		
	n	n	%	MI ^1^	n	%	MI	n	%	MI
*S. aureus*	1	0	0.0	0	1	100.0	0	0	0.0	1
*S. agalactiae*	13	7	70.0	3	2	25.0	6	13	100.0	0
*S. uberis*	11	3	33.3	6	5	62.5	3	8	72.7	3
*Streptococcus* spp.	4	0	0.0	3	2	50.0	2	1	25.0	3
*Staphylococcus* spp.	2	0	0.0	0	1	50.0	1	2	100.0	0
*Enterococcus* spp.	7	3	75.0	1	1	33.3	2	0	0.0	7
*Escherichia coli*	3	0	0.0	1	1	50.0	1	3	100.0	0
Yeasts	3	0	0.0	2	1	100.0	0	2	66.7	1
*Prototheca*	2	0	0.0	2	1	50.0	1	2	100.0	0
**TOTAL**	46	13	41.9	18	15	48.4	16	31	67.4	15

Correctly identified = True Positives + True Negatives; ^1^ MI (misidentified) = False Negatives + False Positives.

**Table 2:** Sensitivity, specificity, and positive predictive value of the magnetic detection and the conventional microbiology, using PCR analysis as the reference method.

	Magnetic Detection		Microbiological Tests
	Anti- *Streptococcus agalactiae*	Anti-GB Streptococci	
True Positives	8	7	31
True Negatives	5	8	0
False Negatives	3	10	0
False Positives	15	6	15
Sensitivity	73%	41%	100%
Specificity	25%	57%	-
^1^PPV	35%	54%	67%

^1^PPV = Positive Predictive Value

## Abbreviations

mAb	monoclonal antibody
pAb	polyclonal antibody
ADC	analogue to digital converter
DC	direct current
Fc fraction	fragment crystallizable region of an immunoglobulin G
GB	Group B
Ig	immunoglobulin
MS	magnetic separation
PBST	phosphate buffer saline with 0.05% ( *v*/*v*) of Tween 20
Oe	Oersted. CGS unit of magnetic field H strength
T	Tesla. SI unit for magnetic induction B strength.
